# Thrombotic Disorders and Antithrombotic Treatments Special Issue

**DOI:** 10.3390/medicina58020229

**Published:** 2022-02-02

**Authors:** Pierpaolo Di Micco

**Affiliations:** Department of Internal Medicine, Fatebenefratelli Hospital of Naples, 80122 Napoli, Italy; pdimicco@libero.it

Thrombotic disorders include a variety of clinical diseases that are common causes of mortality and morbidity in western countries. In this clinical setting, we may include acute coronary syndromes (ACS), acute ischaemic stroke (IS) and pulmonary embolism (PE), although the pathophysiology of venous or arterial thrombosis may be considered very different. In this Special Issue, several authors reported their clinical experiences regarding the diagnostics and therapeutics of several thrombotic diseases, touching on several intriguing points relating to the daily clinical management of thrombotic diseases.

Risk factors for atherothrombosis such as ACS and/or IS, in fact, include prolonged exposition to atherosclerotic diseases such as dyslipidaemia, hypertension, diabetes or exposition to smoking, as the Framingham study identified those predisposing conditions in the XX century. Furthermore, treatments of ACS or IS are based on interventional procedures such as PCI or mechanical thrombolysis that are associated, per se, to further thromboembolic complications [1]. Yet, because ACS and IS are multifactorial diseases, in the last few decades, the association of atherothrombosis to other predisposing conditions as a genetic predisposition was frequently looked for, and inherited thrombophilia was often investigated in this setting without univocal results [2]. So, because the association between inherited thrombophilia and atherothrombosis is still matter of discussion, other genetic alterations that tend to be associated with thrombotic diseases are also under investigation [3]. On the other hand, the association between inherited thrombophilia and thrombotic events is better studied for venous thromboembolism (VTE), and in this setting, the association between gene and environmental causes is well known. Environmental conditions that may predispose one to VTE, in fact, are also indicated as clinical conditions for which pharmacological thromboprophylaxis is always considered, and major orthopaedic surgery is one of them [4,5]. In the last few years, despite the application of international guidelines to prevent VTE after major orthopaedic surgery, the rate of thrombotic complications remained considerable. Several drugs have been tested in this complicated scenario; in particular, subcutaneous injectable anti-Xa inhibitors as unfractionated heparin or low-molecular-weight heparin or fondaparinux and oral anti-Xa inhibitors as direct anticoagulants were tested and are now considered as prophylactic drugs both for elective major orthopaedic surgery and for sudden traumatic orthopaedic surgery [5]. Oral direct anticoagulants are actually the most commonly used drugs, not only for thromboprophylaxis after major orthopaedic surgery, but also in the long-term treatment of VTE. In this way, no differences were found for the long-term anticoagulant treatment of provoked or unprovoked VTE, and they seem to be preferred as they exhibit reduced drug–drug interactions or drug–food interactions in the case of associated comorbidity. Yet, in the presence of VTE-associated comorbidity, the trend to bleeding complications associated with direct oral anticoagulants should be considered, in particular for possible overt or occult gastrointestinal bleedings [6].

Intriguingly, the association between gene–environmental causes that predispose one to VTE is not only on a molecular basis but is also associated with anatomic differences between subject and subject: an anatomic variant of abdominal venous vessels, in fact, has been frequently reported as the main cause of recurrent, unexplained VTE. The left iliac vein is a more commonly known anatomic malformation that causes recurrent, unexplained VTE and is named May Turner syndrome. This anatomic condition is highly relevant during pregnancy and may also explain the increased number of left deep vein thrombosis cases that occur during pregnancy [7]. Of course, both prothrombotic conditions (i.e., the anatomic compression of left iliac vein and pregnancy) interact with all molecular changes that predispose one to a hypercoagulable state and thrombosis during pregnancy [8].

Furthermore, the continuous medical surveillance of old and new diseases in these last 2 years faced a new challenge with regard to the COVID-19 pandemic. Since, the first report from China, an association between COVID-19 and a prothrombotic state has been underlined, and also, an increased rate of VTE events in inpatients was described since first phases of the pandemic. Yet, a confirmed, objective diagnosis of thrombotic events in this setting is always needed, and the best strategy to detect DVT and/or PE in COVID-19 patients early is still matter of discussion [9]; routine screening has been discouraged, but no other strategies have been tested as an effective, alternative method. Furthermore, VTE is not the only thrombotic complication, but also, disseminated intravascular complications may occur with severe complications such as thrombotic microangiopathy, which presents abnormal laboratory findings [10].

In conclusion, we may consider thrombotic disorders to be constant challenges for physicians because they may occur as transversal complications of an underlying disease, and only continuous medical education may offer further improvements from a diagnostic and prognostic point of view.
